# Supplementary material for: Towards unified AI-driven fracture mechanics: the extended deep energy method (XDEM)
Source: Nat Commun. 2026 Aug 15;17:8492. doi: 10.1038/s41467-026-76748-1 (PMC13484471; doi:10.1038/s41467-026-76748-1)
Supplement: Supplementary file 3 [file 41467_2026_76748_MOESM3_ESM.zip › pic/Results/Crack_propagation/Straight/3D_dis_force/Tension_3D/Loss_convergence_10.pdf]

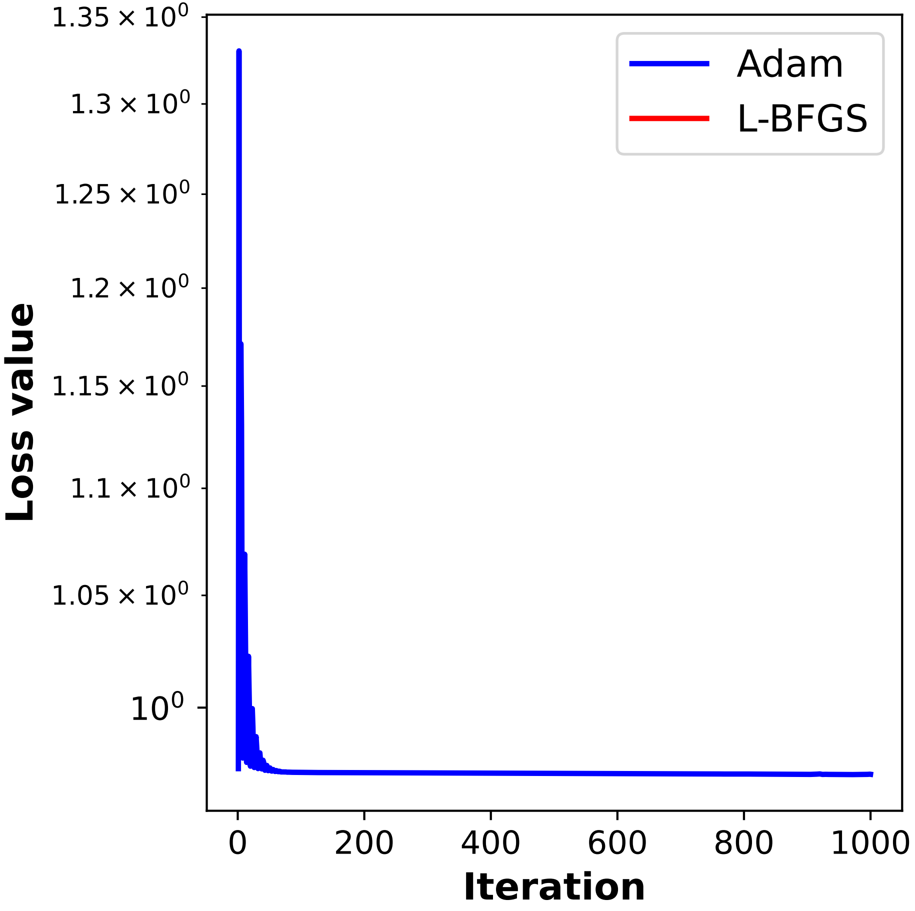

1.35×10^0
1.3×10^0
1.25×10^0
1.2×10^0
1.15×10^0
1.1×10^0
1.05×10^0
10^0
Loss value
0
200
400
600
800
1000
Iteration
Adam
L-BFGS
